# Revisit of Polyaniline as a High-Capacity Organic Cathode Material for Li-Ion Batteries

**DOI:** 10.3390/polym16101401

**Published:** 2024-05-14

**Authors:** Ruirui Zhao, Zu Chang, Xudong Fu, Mingli Xu, Xinping Ai, Jiangfeng Qian

**Affiliations:** 1 Research Institute, EVE Battery Corporation Limited, Huizhou 516006, China; zhaoruirui@evebattery.com; 2 College of Chemistry and Molecular Sciences, Wuhan University, Wuhan 430072, China; 2023102030016@whu.edu.cn (Z.C.); 2021202030102@whu.edu.cn (M.X.); xpai@whu.edu.cn (X.A.); 3 New Materials and Green Manufacturing Talent Introduction and Innovation Demonstration Base, Hubei Provincial Key Laboratory of Green Materials for Light Industry, Hubei University of Technology, Wuhan 430068, China

**Keywords:** organic cathode material, polyaniline, Li-ion battery, high electrochemical utilization, oxidative degradation

## Abstract

Polyaniline (PANI) has long been explored as a promising organic cathode for Li-ion batteries. However, its poor electrochemical utilization and cycling instability cast doubt on its potential for practical applications. In this work, we revisit the electrochemical performance of PANI in nonaqueous electrolytes, and reveal an unprecedented reversible capacity of 197.2 mAh g^−1^ (244.8 F g^−1^) when cycled in a wide potential range of 1.5 to 4.4 V vs. Li^+^/Li. This ultra-high capacity derives from 70% -NH- transformed to =NH^+^- during deep charging/discharging process. This material also demonstrates a high average coulombic efficiency of 98%, an excellent rate performance with 73.5 mAh g^−1^ at 1800 mA g^−1^, and retains 76% of initial value after 100 cycles, which are among the best reported values for PANI electrodes in battery applications.

## 1. Introduction

Compared carbon materials (carbon nanotubes, graphene) [1,2], organic electrode materials, such as conducting polymers (polyaniline, polypyrrole, polythiophene), carbonyl compounds (quinones, dianhydrides), and free radical compounds are fascinating candidates for the next-generation energy storage applications because of their cost-effective, structural diversity and materials sustainability [3,4,5,6]. The carbonyl compounds and free radical compounds can be dissolved in electrolytes easily, resulting in low cycle stability. Conducting polymers consists of large molecules by covalent bonds, which increase resistance to dissolve in electrolytes and cycle stability. Among conducting polymers, polyaniline (PANI) has been actively explored since its first discovery by MacDiarmid in the early 1980s because of its high conductivity, good electrochemical and environmental stability, and interesting redox behavior [7,8,9]. The electrochemical reactions of PANI electrodes proceed through the redox conversions between diaminobenzenoid and diiminoquinoid rings, accompanying the reversible doping/dedoping of electrolyte anions into/from the PANI cathode. The theoretical capacity of PANI would be 295 mAh g^−1^ [10]. In addition, benefitting from its high electronic conductivity, PANI allows ultra-fast ion-insertion kinetics comparable to that of supercapacitors, thus enabling it to be a promising high capacity and high-power cathode material for Li-ion batteries.

However, the practical application of PANI in Li-Ion batteries has been hindered by its poor electrochemical utilization and cycling instability [11]. For example, earlier studies found that PANI materials, either synthesized via electropolymerization or chemical polymerization methods, could only deliver a reversible capacity of around 120 mAh g^−1^ in the potential range of 1.5 to 3.8 V vs. Li^+^/Li [12,13]. This means that less than half of the redox groups are electroactive during charging/discharging process. When PANI electrodes were charged to a much higher cut-off potential of 4.3 V vs. Li^+^/Li, an unprecedented initial charge capacity of 270 mAh g^−1^ was first reported by Haas et al. [14], but only 160 mAh g^−1^ could be realized during the initial discharge process and decreased rapidly in the following cycles. This massive capacity loss was attributed to the degradation of the polymer backbone as well as the decomposition of the organic electrolyte by overoxidation at high voltage [15]. In order to improve the capacity utilization and stability of PANI electrode, various advanced nanostructures [16,17,18] and molecular designs [19,20] had been reported. For instance, Yi and coworkers [17] developed a sacrificial template method to obtain PANI nanowire arrays, which showed a discharge capacity of 160 mAh g^−1^ and retained 75% of initial value in the 100 cycles. Yang et al. [19] prepared an aniline/o-nitroaniline copolymer by introducing an electron drawing group o-nitroaniline onto the PANI chains. This material achieved a greatly improved discharge capacity of 181 mAh g^−1^ at the first cycle and remained 173 mAh g^−1^ after 50 cycles. Nevertheless, judging from the research status and achievements by far, we can find that the potential of PANI material has not yet been fully exploited. There still has vast research space to improve reversible capacity of PANI for battery applications.

Herein, the reversible capacity of PANI is improved by a simple method, which is optimizing the potential window. We revisit the electrochemical performance of PANI material in nonaqueous Li-ion batteries and reveal for the first time an unprecedented reversible capacity of 197.2 mAh g^−1^ (244.8 F g^−1^) when cycled between 1.5 and 4.4 V (vs. Li^+^/Li, all electrode potentials are referenced to Li+/Li in this work), corresponding to about 70% theoretical capacity of PANI. This material also demonstrates a high average coulombic efficiency of 98%, an excellent rate performance with 73.5 mAh g^−1^ at 1800 mA g^−1^, and quite good cycle stability with 76% retained after 100 cycles at 20 mA g^−1^, which are among the best reported values for PANI materials used in Li-ion batteries.

## 2. Materials and Methods

Materials synthesis: Polyaniline (PANI) was synthesized by chemical oxidative polymerization. Firstly, 1.86 g aniline and 4.56 g ammonium peroxydisulfate were dissolved in 100 mL HCl aqueous solution (1 mol L^−1^), respectively. The two solutions were cooled to 0–4 C and mixed. The polymerization was carried out for 24 h at 0–4 C. Then, the PANI was filtrated in a vacuum and washed several times with ultrapure water and NH_3_ aqueous solution (1 mol L^−1^). Finally, the PANI was dried at 60 C for 12 h, and used as active material of lithium-ion batteries.

Morphological and structural characterizations: Scanning electron microscope (SEM, SU8010, Hitachi, Tokyo, Japan) equipped with an energy dispersive spectrometer (EDS, X-MAX, Oxford, Oxford, UK) was utilized to observe the morphology and the element of the PANI. The crystalline structure of the PANI was characterized by X-ray diffraction (XRD, XRD-6000, Shimadzu, Shanghai, China) with Cu-Kα radiation in the range from 10° to 80°. Fourier transform infrared spectroscopy (FTIR, TENSOR II, Bruker Optics Inc. Billerica, MA, USA) was used to obtain vibrational spectra of the PANI.

Electrochemical measurements: Electrochemical performance of the PANI was carried out using 2032-type coin cells. The cathode was made by pressing a 0.8 cm^2^ PANI film onto an aluminum mesh. The PANI film electrode was prepared by rolling a mixture containing 70 wt.% PANI, 20 wt.% Super P and 10 wt.% polytetrafluoroethylene. The mass loading of the PANI film electrode was 2.5–3.0 mg cm^−2^. The anode was a disk of lithium metal foil, and the electrolyte was 1 mol L^−1^ LiClO_4_ dissolved in ethylene carbonate/diethyl carbonate (1:1, by volume). A microporous membrane (Celgard 2500) was used as a separator. The batteries were assembled in a dry box filled with argon gas and tested at room temperature. Cyclic voltammetry (CV) was carried out at a scan rate of 0.1 mV s^−1^ using a CHI 660a electrochemical workstation (Chenhua Instruments Co., Wuhan, China). Before recording the CV curves, the PANI film electrode was cleaned by 5 potential cycles (100 mV s^−1^) between 1.5 and 4.0 V. A galvanostatic charge/discharge (GCD) test was performed on a LAND CT2001 battery tester (Wuhan LAND Electronic Co. Ltd., Wuhan, China). The PANI at a fully charged state after 50 GCD cycles (1.5 A g^−1^) in different potential ranges were used for ex-situ FTIR test.

## 3. Results and Discussion

**Figure 1:**
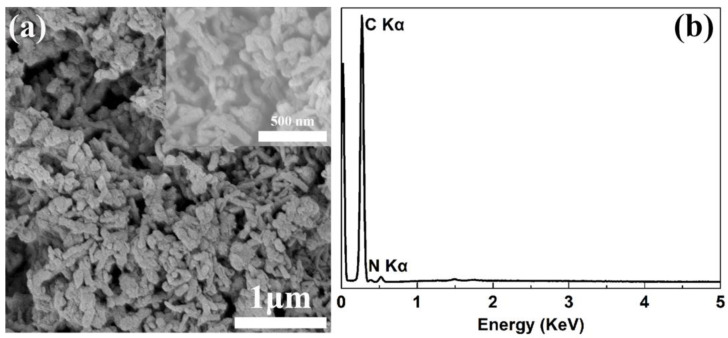
Scanning electron microscope image with different magnification (**a**) and energy dispersive X-ray spectroscopy curve (**b**) of the dedoped PANI.

PANI was synthesized by oxidative polymerization of aniline monomers using (NH_4_)_2_S_2_O_8_ as an oxidant in a HCl acid media, followed by deprotonation and/or dedoping treatment with NH_3_ aqueous solution (1 mol L^−1^) to yield the final product. Elemental analysis indicates that the C, N, and H contents are 79.39, 15.27, and 4.82 wt.% in the dedoped PANI, respectively, which are consistent with theoretical values of PANI (Table S1). As shown in Figure 1a, the as-prepared PANI powders emerge as aggregated nanorods with an average length of ~200 nm. Energy dispersive X-ray spectroscopy (EDS) and X-ray photoelectron spectroscopy (XPS) show that only carbon and nitrogen elements are present in the PANI sample (Figure 1b and Figure S1), while no sulfur and chlorine can be detected, indicating the PANI is fully dedoped. The dedoping process is shown in Figure S2a. The deconvoluted N1s XPS spectrum in Figure S2b reveals two characteristic peaks belong to quinoid imine (-N=) and benzenoid amine (-NH-) units at 397.3 eV and 398.4 eV, respectively. The intensity ratio of the two peaks is about 0.7:0.3 [21,22]. The X-ray diffraction pattern (XRD) in Figure S3 clearly exhibits a broad diffuse scattering with a maximum at about 19°, suggesting that PANI sample is in amorphous phase [23].

**Figure 2:**
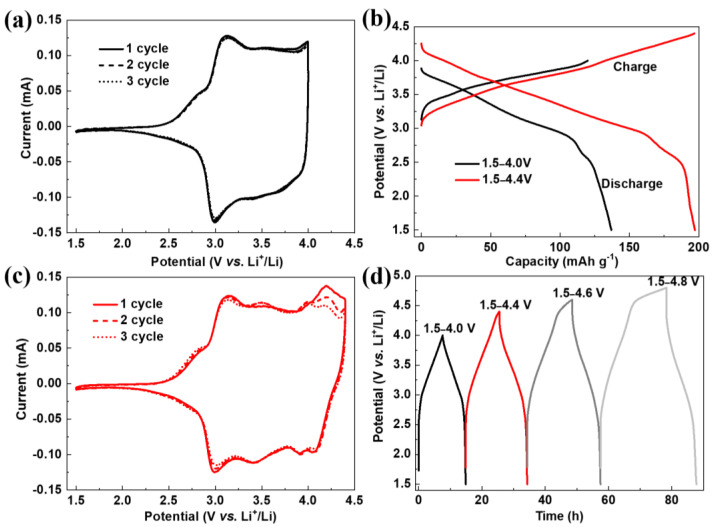
(**a**,**c**) Cyclic voltammetry curves of PANI electrode at 0.1 mV s^−1^ in 1.5–4.0 V (**a**) and 1.5–4.4 V (**c**); (**b**,**d**) Galvanostatic charge/discharge (GCD) curves at 20 mA g^−1^ (0.1 C, 1 C = 200 mA g^−1^) in different potential ranges.

**Figure 3:**
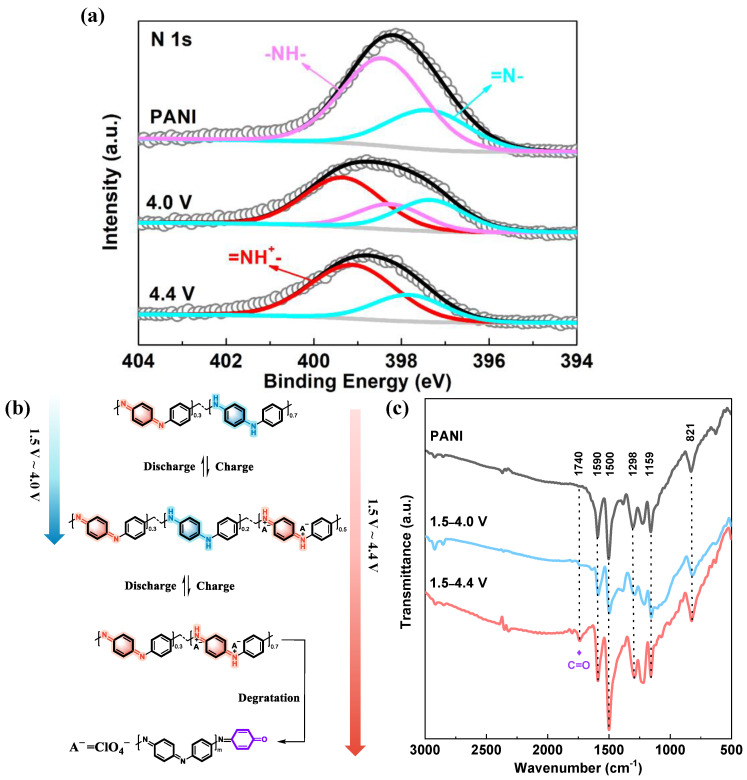
(**a**) N1s XPS spectrum of the PANI film electrode at its initial state, 4.0 V and 4.4 V (the red line: =NH^+^–; the purple line: –NH–; the blue line: =N–); (**b**) schematic illustration of reversible redox reactions between different PANI forms and PANI degradation during the electrochemical process at high potential; (**c**) FTIR of the as-prepared PANI and PANI electrodes after 50 GCD cycles in different potential ranges.

When 1 mol L^−1^ LiClO_4_/ethylene carbonate/diethylcarbonate is used as an electrolyte, the PANI is insoluble in the electrolyte [24]. Figure 2a shows the cyclic voltammetry (CV) curves of the PANI electrode in the potential range of 1.5 to 4.0 V. The main feature in the CV curves is one pair of broad and symmetric redox bands at around 3.0 V, which can be attributed to reversible transition between benzenoid amine (-NH-) and protonated quinoid imine (=NH^+^-) [22,25]. In addition, the shapes and areas of the redox bands remain almost unchanged during successive scans, suggesting a highly reversible electrochemical reaction occurring at this potential interval. Galvanostatic charge/discharge (GCD) curves for the PANI electrode are present in Figure 2b. The PANI electrode delivers an initial discharge capacity of 150.9 mAh g^−1^ (217.3 F g^−1^) within 1.5–4.0 V, which is approximate half theoretical capacity of PANI. To research the PANI changes in the discharge process, the N1s XPS spectra of PANI electrode at its initial state and 4.0 V are tested (Figure 3a). Compared to initial state of PANI, about 50% -NH- transforms to =NH^+^- at 4.0 V (Table 1) [22], which is consistent with 150.9 mAh g^−1^ discharge capacity within 1.5–4.0 V (half theoretical capacity of PANI).

In order to achieve higher electrochemical utilization, we elevated the charge cut-off potential to a high value of 4.4 V. As shown in Figure 2c, a new pair of redox peaks occurs at around 4.2 V, which may arise from the PANI degradation [26,27]. Although all benzenoid amine (-NH-) in the PANI can be consecutively activated to protonated quinoid imine (=NH^+^-), the PANI with all quinoid structure is not chemically stable because of its susceptibility to be nucleophilic attacked by organic electrolyte [28]. The deep charge process is supposed to be accompanied by serious structural damage, i.e., the oxidative collapse of the polymer backbone to generate oligoanilines [28,29]. CV curves in Figure 2c also indicate that the intensity of the oxidation peak at 4.2 V decreases in the subsequent scans, manifesting the gradual deterioration of the PANI under high operating potential. The initial discharge capacity is improved to 197.2 mAh g^−1^ (244.8 F g^−1^) within 1.5–4.4 V (Figure 2b), corresponding to about 70% theoretical capacity of PANI, which is consistent 70% -NH- transforms to =NH^+^- at 4.4 V compared to initial state of PANI (Figure 3a and Table 1) [22].

Further elevating cut-off potentials in excess of 4.4 V (Figure 2d), we can observe continuously increased charge capacities to 326.7 and 479.4 mAh g^−1^ at 4.6 and 4.8 V, respectively, much higher than that obtained at 1.5–4.4 V. But the discharge capacities are almost equal at the three cut-off potentials. The extra charge capacities mainly derive from the irreversible decomposition of the organic electrolyte at potentials higher than 4.4 V [30,31]. Therefore, the upper cut-off potential is set as 4.4 V concerning the trade-off between capacity and reversibility.

The working mechanism for the potential-dependent capacity release is schematically summarized in Figure 3b. The 20% diiminoquinoid rings of PANI are electrochemical inertness, which remain unchanged during charging/discharging process. Compared, only 50% -NH- transformed to =NH^+^- between 1.5 and 4.0 V; 70% -NH- transformed to =NH^+^- in 1.5–4.4 V delivers higher capacity. But the PANI is degraded during the electrochemical cycle at 1.5–4.4 V. The degradation products of PANI are hydroxyl- or amino-terminated oligoanilines in aqueous electrolytes [26,32,33]. Thus, it is rational to infer that the degradation products in organic electrolytes are carbonyl-terminated oligoanilines because of the lack of proton. Ex-situ fourier transform infrared spectroscopy (FTIR, Figure 3c) is further employed to support the hypothetical mechanism. The as-prepared PANI sample shows clearly characteristic vibrations of diiminoquinoid rings (1590 cm^−1^), diamino-benzenoid rings (1500 cm^−1^), C-N (1298 cm^−1^), and C-H (1159 and 821 cm^−1^), which are consistent the previously reported spectra of PANI [23,34]. After 50 GCD cycles between 1.5 and 4.0 V, all the characteristic peaks are reserved, in agreement with the good reversibility within this potential range (Figure 2a). By stark contrast, a new band at 1740 cm^−1^ appears after being cycled from 1.5 to 4.4 V, which is assigned to the stretching vibration of the carbonyl group [35]. These results provide strong evidence for the hypothetical mechanism.

**Figure 4:**
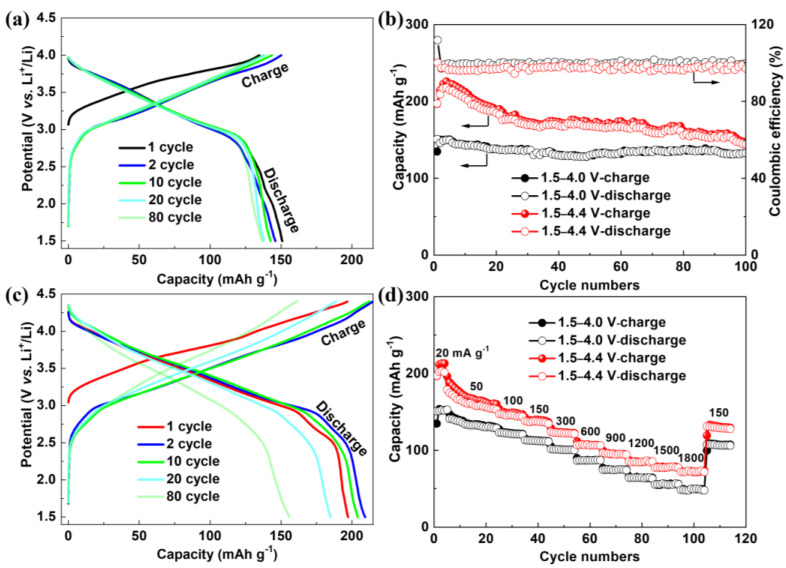
(**a**,**c**) Galvanostatic charge/discharge curves of the PANI between 1.5 and 4.0 V (**a**) and 1.5–4.4 V (**c**) at 20 mA g^−1^ (0.1 C); (**b**,**d**) Cycle performances at 20 mA g^−1^ (**b**) and rate performances from 0.1 C to 9 C (**d**) of the PANI in different potential ranges.

Figure 4a–c presents the long-term cycling stability and rate performances of PANI electrodes in different potential ranges. In accord with the CV data, the potential profiles keep stable with almost no discernible capacity decay when cycled between 1.5 and 4.0 V (Figure 4a). The reversible capacity is about 150 mAh g^−1^ and retains 90% of initial value after 100 cycles (Figure 4b), demonstrating an excellent cycle stability of PANI electrode if only 50% -NH- transformed to =NH^+^-. When cycled in the wider potential range of 1.5 to 4.4 V, the discharge capacity first slightly increases from 197.2 mAh g^−1^ to a maximum value of 217.8 mAh g^−1^ in the initial 4 cycles (Figure 4b), corresponding to a nearly 45% increase compared with that obtained at 1.5–4.0 V, which is a quite outstanding value for PANI electrodes in the light of previous reports (Table S2). However, the reversible capacity gradually fades and the cell overpotential increases during subsequent cycles (Figure 4c), possibly caused by the deterioration of the PANI under high operating potential as evidenced above. Nevertheless, the PANI electrode still retains a reversible capacity of 148.9 mAh g^−1^ with a capacity retention of 76% after 100 cycles (Figure 4b). Moreover, the PANI delivers a high average coulombic efficiency of 97.5% and 98.0% at 100 and 200 cycles, respectively (Figure S4).

The rate performances of PANI were investigated from 20 to 1800 mA g^−1^ (Figure 4d). The reversible capacities in the potential range of 1.5–4.4 V are superior to those obtained in 1.5–4.0 V at all rates. At a low rate of 20 mA g^−1^, the capacities have the same trend as the cycle tests (Figure 4b), and then decrease slightly at 50 mA g^−1^, whereas they remain stable at higher rates. In 1.5–4.0 V, the PANI displays discharge capacities of 113.3, 75.2, and 50.0 mAh g^−1^ at the current densities of 150, 900, and 1800 mA g^−1^, respectively. In the wider potential range of 1.5–4.4 V, these values are much higher, that is, 138.3, 96.7, and 73.5 mAh g^−1^, respectively. Moreover, when the current density is changed back to 150 mA g^−1^, the discharge capacity can be mostly recovered even after 105 cycles (107.7 mAh g^−1^ in 1.5–4.0 V and 131.2 mAh g^−1^ in 1.5–4.4 V), indicating high-rate tolerance of the PANI cathode. The cycle performances of PANI at high rates were also tested (Figures S5 and S6). At 150 mA g^−1^, the PANI achieves stable discharge capacities of 107.8 and 131.2 mAh g^−1^ in 1.5–4.0 V and 1.5–4.4 V, respectively. Impressively, the PANI displays a discharge capacity of 72.9 mAh g^−1^ with a capacity retention of almost 100% at 1500 mA g^−1^ from 1.5 to 4.4 V (Figure S6).

## 4. Conclusions

In summary, PANI material was synthesized by chemical oxidative polymerization and employed as an organic cathode for the Li-ion battery. This material shows a highly reversible capacity of 150.9 mAh g^−1^ (217.3 F g^−1^) in 1.5–4.0 V, corresponding to about 50% theoretical capacity of PANI. When the upper cut-off potential is elevated to 4.4 V, the PANI electrode delivers an unprecedented reversible capacity of 197.2 mAh g^−1^ (244.8 F g^−1^), corresponding to about 70% theoretical capacity of PANI, which is among the best reported values for PANI materials in Li-ion batteries. The extra capacity derives from all -NH- in dedoped PANI transformed to =NH^+^- in 1.5–4.4 V. This work provides a simple method to improve the capacity of PANI and is expected to promote the development of Li-ion batteries based on organic electrode materials.

## Figures and Tables

**Table 1:** The =N-, -NH-, and =NH+- contents of the PANI film electrode at its initial state, 4.0 V and 4.4 V.

	=N-	-NH-	=NH^+^-
PANI	30.02%	69.98%	-
4.0 V	28.90%	21.70%	49.39%
4.4 V	29.06%	-	70.94%

## Data Availability

Data are contained within the article and Supplementary Materials.

## Supplementary Materials

The following supporting information can be downloaded at: https://www.mdpi.com/article/10.3390/polym16101401/s1, Figure S1. XPS spectrum of the dedoped PANI sample. Figure S2. (a) Structure characterization of PANI before and after dedoping. (b) N1s XPS spectrum of the dedoped PANI. Figure S3. XRD curve of the dedoped PANI. Figure S4. Cycling performance at 0.02 A g−1 of PANI in potential range of 1.5 to 4.4 V. Figure S5. Cycling performance of PANI at 0.15 A g−1 (a) and 1.5 A g−1 (b) in potential range of 1.5 to 4.0 V. Figure S6. Cycling performance of PANI at 0.15 A g−1 (a) and 1.5 A g−1 (b) in potential range of 1.5 to 4.4 V. Table S1. Elemental analysis data (wt%) of as synthesized PANI powder and dedoped PANI powder. Table S2. A survey of the electrochemical performances (including reversible capacity, coulombic efficiency and cycling stability) of PANI electrodes reported in literatures. References [36,37,38,39,40,41,42] are cited in the Supplementary Materials.
