# Supplementary material for: Revisit of Polyaniline as a High-Capacity Organic Cathode Material for Li-Ion Batteries
Source: Polymers (Basel). 2024 May 14;16(10):1401. doi: 10.3390/polym16101401 (PMC11124868; doi:10.3390/polym16101401)
Supplement: Supplementary file 1 [file polymers-16-01401-s001.zip › polymers-2971688-supplementary.pdf]

Electronic Supplementary Materials

Revisit of Polyaniline as A High-Capacity Organic Cathode Material for Li-Ion Batteries

Ruirui Zhao [1,2,†], Zu Chang [2,†], Xudong Fu [2,3,*], Mingli Xu [2], Xinping Ai [2] and Jiangfeng Qian [2,*]

[1] Research Institute, EVE Battery Corporation Limited, Huizhou 516006, China; zhaoruirui@evebattery.com
[2] College of Chemistry and Molecular Sciences, Wuhan University, Wuhan 430072, China; 2023102030016@whu.edu.cn (Z.C.); 2021202030102@whu.edu.cn (M.X.); xpai@whu.edu.cn (X.A.)
[3] New Materials and Green Manufacturing Talent Introduction and Innovation Demonstration Base, Hubei Provincial key Laboratory of Green Materials for Light Industry, Hubei University of Technology, Wuhan 430068, China
* Correspondence: fuxudong@hbut.edu.cn (X.F.); jfqian@whu.edu.cn (J.Q.)
† These authors contributed equally to this work.

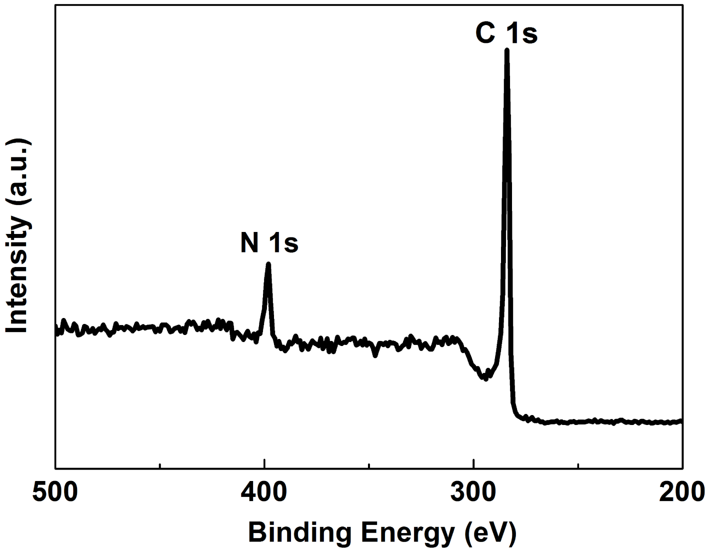

Figure S1. XPS spectrum of the dedoped PANI sample.

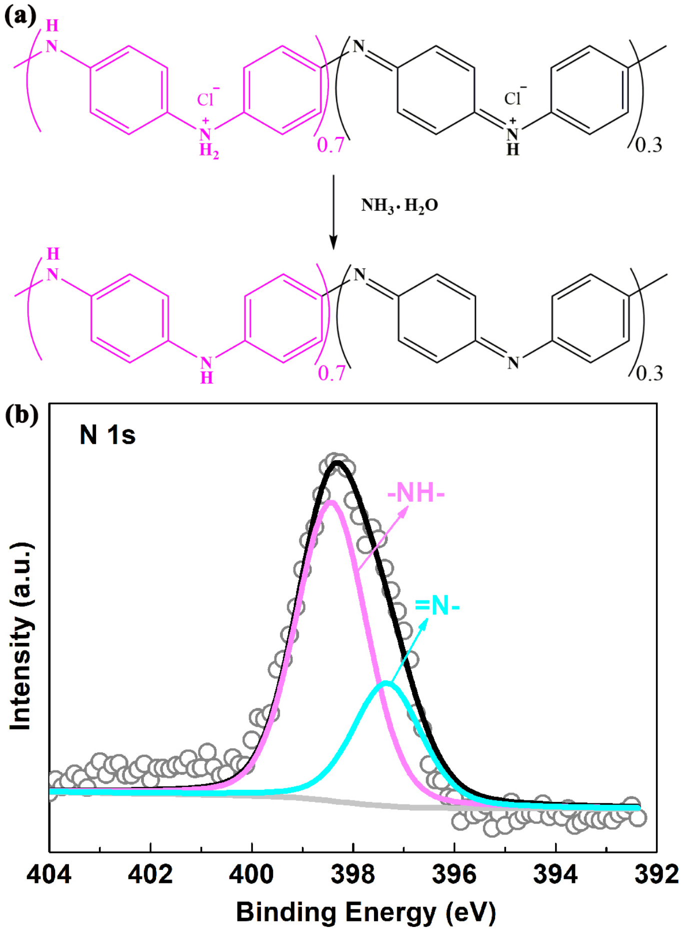

Figure S2. (**a**) Structure characterization of PANI before and after dedoping. (**b**) N1s XPS spectrum of the dedoped PANI.

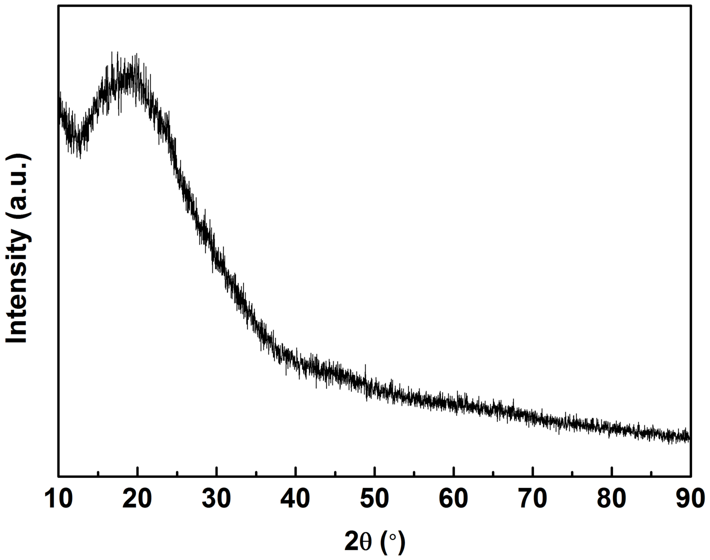

Figure S3. XRD curve of the dedoped PANI.

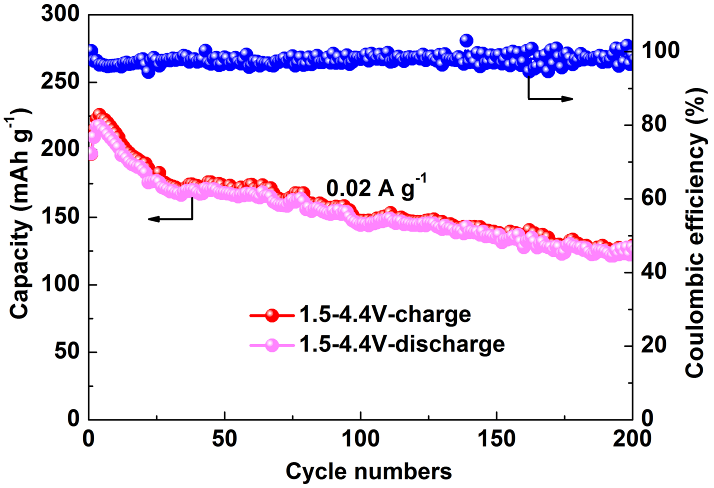

Figure S4. Cycling performance at 0.02 A g^{-1} of PANI in potential range of 1.5 to 4.4 V.

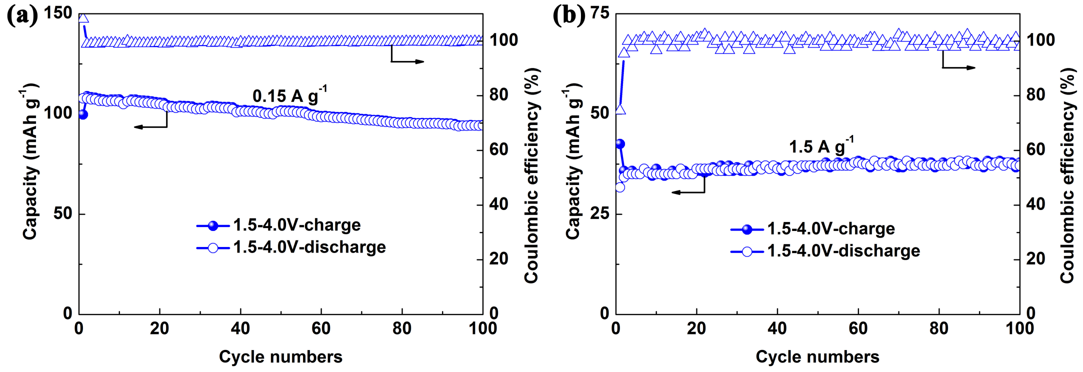

Figure S5. Cycling performance of PANI at 0.15 A g^{-1} (**a**) and 1.5 A g^{-1} (**b**) in potential range of 1.5 to 4.0 V.

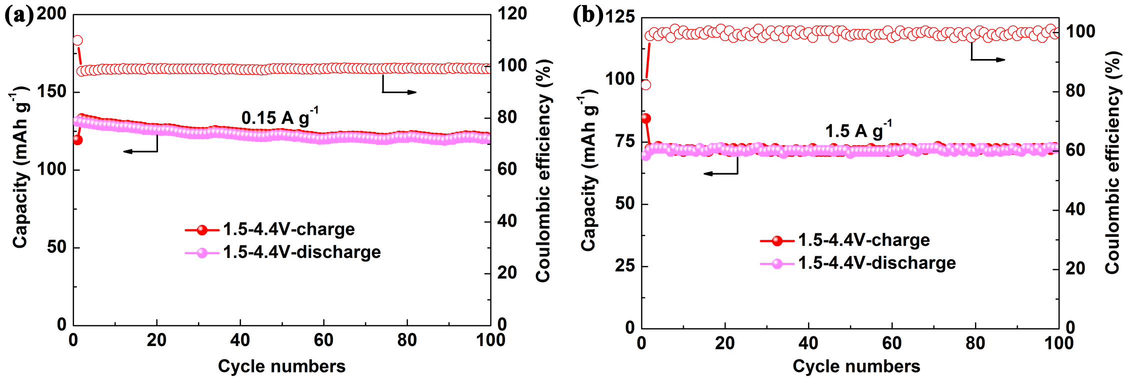

Figure S6. Cycling performance of PANI at 0.15 A g^{-1} (**a**) and 1.5 A g^{-1} (**b**) in potential range of 1.5 to 4.4 V.

Table S1. Elemental analysis data (wt%) of as synthesized PANI powder and dedoped PANI powder.

Element	C (wt.%)	N (wt.%)	H (wt.%)
Synthesized PANI	56.98	10.75	5.18
Dedoped PANI	79.39	15.27	4.81

The synthesized PANI is Cl-doped PANI. The Cl content in the synthesized PANI is 27.09 wt.% (100-56.98-10.75-5.18=27.09). The molar ratio of N and Cl is 1:1 in the synthesized PANI, so the PANI is fully doped by Cl. The C, N, and H contents are 79.39, 15.27 and 4.82 wt.% in the dedoped PANI, respectively, which are consistent with theoretical values of PANI, so the PANI is fully dedoped.

Table S2. A survey of the electrochemical performances (including reversible capacity, coulombic efficiency and cycling stability) of PANI electrodes reported in literatures.

Sample	Potential Range (V vs. Li/Li^+)	Capacity (mAh g^{-1})	Coulombic Efficiency	Cycling Performance	Ref.
PANI film	2.0-3.9	50 (0.05 mA cm^{-2})	-	30 cycles	S2
PANI film	2.4-3.8	107 (0.02 mA cm^{-2})	97.5%	12 cycles	S3
PANI nanotubes	2.0-3.9	75.7 (20 mA g^{-1})	94.9-97.5%	72.3 mAh g^{-1} after 80 cycles	S4
PANI nanofibers	1.5-4.2	133.5 (0.1 C)	-	82.5 mAh g^{-1} after 50 cycles	S5
Mechanochemically prepared PANI	2.0-4.2	145 (15 mA g^{-1})	-	130 cycles	S6
Electrosprayed PANI	1.5-4.0	142.5 (0.1 C)	-	86.5 mAh g^{-1} after 50 cycles	S7
PANI nanowire arrays	2.0-4.0	159.83 (30 mA g^{-1})	about 100%	119.79 mAh g^{-1} after 100 cycles	S8
Reduced PANI	2.2-4.2	96.1 (0.2 C)	>90%	78.0 mAh g^{-1} after 100 cycles	S9
PANI	2.5-4.3	130 (100 mA g^{-1})	>99%	200 cycles	S10
PANI	1.5-4.0	141 (0.1 C)	97%	120 mAh g^{-1} after 40 cycles	S11

Lithium n-Doped PANI	2.5-4.25	230 (0.1 C)	>99%	400 cycles	S12
PANI	2.0-4.2	50	84%	246 mAh g^{-1} after 2000 cycles	S13
**PANI**	**1.5-4.0**	**150.9 (20 mA g^{-1})**	**>98%**	**133.7 mAh g^{-1} after 100 cycles**	**This work**
**PANI**	**1.5-4.4**	**217.8 (20 mA g^{-1})**	**>96%**	**145.1 mAh g^{-1} after 100 cycles**	

S1. Xie, J.; Gu, P.; Zhang, Q. *ACS Energy Lett.* **2017**, *2*, 1985–1996.

S2. Ryu, K.S.; Kim, K.M.; Kang, S.-G.; Lee, G.J.; Joo, J.; Chang, S.H. *Synth. Met.* **2000**, *110*, 213–217.

S3. Venancio, E.C.; Motheo, A.J.; Amaral, F.A.; Bocchi, N. *J. Power Sources* **2001**, *94*, 36–39.

S4. Cheng, F.; Tang, W.; Li, C.; Chen, J.; Liu, H.; Shen, P.; Dou, S. *Chem. Eur. J.* **2006**, *12*, 3082–3088.

S5. Yang, H.; Song, T.; Liu, L.; Devadoss, A.; Xia, F.; Han, H.; Park, H.; Sigmund, W.; Kwon, K.; Paik, U. *J. Phys. Chem. C* **2013**, *117*, 17376–17381.

S6. Posudievsky, O.Y.; Kozarenko, O.A.; Dyadyun, V.S.; Koshechko, V.G.; Pokhodenko, V.D. *Synth. Met.* **2012**, *162*, 2206–2211.

S7. Manuel, J.; Raghavan, P.; Shin, C.; Heo, M.-Y.; Ahn, J.-H.; Noh, J.-P.; Cho, G.-B.; Ryu, H.-S.; Ahn, H.-J. *Mater. Res. Bull.* **2010**, *45*, 265–268.

S8. Li, X.; Wu, Y.; Hua, K.; Li, S.; Fang, D.; Luo, Z.; Bao, R.; Fan, X.; Yi, J. *Colloid. Polym. Sci.* **2018**, *296*, 1395–1400.

S9. Liu, P.; Han, J.-J.; Jiang, L.-F.; Li, Z.-Y.; Cheng, J.-N. *Appl. Surf. Sci.* **2017**, *400*, 446–452.

S10. Rauhala, T.; Davodi, F.; Sainio, J.; Sorsa, O.; Kallio, T. *Electrochim. Acta* **2020**, *336*, 135735.

S11. Manuel, J.; Kim, J.-K.; Matic, A.; Jacobsson, P.; Chauhan, G.S.; Ha, J.K.; Cho, K.-K.; Ahn, J.-H. *Mater. Res. Bull.* **2012**, *47*, 2815–2818.

S12. Jiménez, P.; Levillain, E.; Alévêque, O.; Guyomard, D.; Lestriez, B.; Gaubicher, J. *Angew. Chem. Int. Edit.* **2017**, *56*, 1553–1556.

S13. Guo, Z.; Wang, J.; Yu, P.; Li, M.; Huang, L.; Hu, Z.; Wang, Y.; Song, Z. *Adv. Energy Mater.* **2023**, *13*, 2301520–2301531.
